# Congenital eye malformations and their impact on the health status of the Mexican population

**DOI:** 10.3389/fmed.2025.1685332

**Published:** 2025-11-06

**Authors:** Iván Antonio García-Montalvo, María José Ruiz Sanginéz, Marco Antonio Sánchez-Medina, Enrique López-Ramírez, Carlos Francisco Varapizuela-Sánchez, Diana Matías-Pérez

**Affiliations:** 1 División de Estudios de Posgrado e Investigación, Tecnológico Nacional de México/Instituto Tecnológico de Oaxaca, Oaxaca, México; 2 Departamento de Ingeniería Química, Tecnológico Nacional de México/Instituto Tecnológico de Oaxaca, Oaxaca, México

**Keywords:** congenital eye malformations, early diagnosis, public policy, public health systems, Mexico

## 1 Introduction

Congenital ocular malformations are a set of structural abnormalities present from birth that affect eye development, with severe consequences for vision and, therefore, for the individual's quality of life (1, 2). In Mexico, these pathologies represent a public health problem that goes beyond the clinical sphere and has a significant impact on social, educational, and economic spheres (3, 4). The visual impairment they cause not only limits the functionality of those affected, but also places a significant burden on families and the public health system, many of these families having limited resources to cope with these challenges.

Unfortunately, despite the importance of these diseases, Mexico faces a considerable lack of up-to-date epidemiological data and insufficient efforts to establish early diagnosis and effective prevention programs. This hinders the design and implementation of adequate public policies (5). It is crucial to integrate political participation to promote effective policies in this area. Malformations encompass various pathologies that affect different parts of the eye, including microphthalmia, anophthalmia, colobomas, anterior segment disorders, congenital cataracts, eyelid abnormalities, and congenital tumors such as retinoblastoma (6–8). The incidence varies according to regions of the country and the degree of access to specialized services, reflecting existing inequalities in the Mexican health system.

Effective diagnosis requires a comprehensive approach that combines detailed clinical examinations and imaging tests, complemented in many cases by genetic testing to identify mutations associated with congenital syndromes, such as next-generation sequencing (3, 9). This genetic diagnosis is crucial for planning reproduction, detecting comorbidities, and guiding appropriate therapies, benefiting both the patient and their family members. However, the lack of technological infrastructure and qualified personnel significantly limits early detection and, therefore, the possibilities for intervention that can improve visual function.

Ocular malformations arise from the complex interaction between genetic and environmental factors. At the genetic level, mutations in genes such as *FOXE3, PAX*6, and *SOX2* play a fundamental role and can be hereditary, *de novo*, or part of multisystemic conditions, complicating diagnosis and clinical management (10–12). In terms of environmental factors, significant risks persist in Mexico, such as exposure to viruses such as toxoplasmosis, rubella, or cytomegalovirus, consumption of teratogenic substances, malnutrition, and folic acid deficiency. In addition, socioeconomic precariousness in various Mexican communities increases the vulnerability of pregnant women to these factors (6, 13).

Another critical issue is the lack of up-to-date data on the prevalence of these malformations in Mexico, which prevents the creation of evidence-based public health policies. Unlike other countries that have national surveillance and neonatal screening systems, such as the EUROCAT program in Europe (14), Mexico lacks a systematic national registry that would allow for the analysis and evaluation of the frequency and types of congenital malformations at the national level. Some local efforts have generated hospital databases that report varying prevalences, but without achieving national coverage or continuity (15, 16).

It should be noted that congenital eye malformations are not due to a single identifiable cause, but rather to a combination of various factors. These pathologies must be addressed through a multidisciplinary approach to strengthen prevention and thereby mitigate them. The objective of this article is to jointly analyze congenital eye malformations present in Mexico, from the point of view of their causes, as well as the diagnosis used, the impact on the Mexican health system, and the direct effect they may have on society, in addition to the importance of political participation in establishing a series of public policies that support care and seek to improve the quality of life of affected individuals and their families.

## 2 Systemic impact of congenital eye malformations on health status and Mexican society

Prenatal care is the maternal care received throughout pregnancy, aiming to preserve and improve health and ensure the pregnancy reaches full term in the best possible way. This care is one of the fundamental components of maternal health; however, the quality of this service and prenatal care rates vary between countries, with developed countries having high rates and developing countries having the lowest. This reflects the increase in maternal and neonatal mortality in developing countries. Women who receive prenatal care have lower maternal and infant mortality rates, as well as better pregnancy outcomes, and the use of prenatal care correlates with higher average birth weight and greater gestational age. Since prenatal care is provided by the health system, it should be available to all women during pregnancy. However, this is not the case, as there are conditions that prevent pregnant women from attending prenatal checkups. This is related to socioeconomic, sociodemographic, and even cultural limitations, which hurt prenatal care (17, 18).

These malformations not only hurt the lives of those who suffer from them, but also their families, who may experience some form of social discrimination. The visual impairment caused by these conditions varies, ranging from mild reduction to total blindness, thereby affecting the motor, cognitive, and emotional development of the individual from an early age (19). From the perspective of the health system, congenital eye malformations pose a significant challenge for pediatric ophthalmological care. Resources are needed to establish the correct diagnosis, followed by surgical treatment, visual rehabilitation, and psychosocial support for the patient and their families. In the medical field in Mexico, the lack of specialists, poor and limited hospital infrastructure, and uneven geographical distribution of medical services significantly contribute to patients not receiving the timely care they require.

Children with visual impairments face complex barriers that hinder their access to inclusive, quality education, thereby affecting their future employment opportunities. These barriers stem not only from personal limitations but are also profoundly influenced by structural, social, and cultural factors (20). In Mexico, special education has been focused on the basic level, which includes preschool, primary, and secondary education, with the creation of institutions that teach essential knowledge through specific support systems, such as the Braille system for the blind and visually impaired, or Mexican sign language for people with speech and hearing impairments. However, inclusive education faces significant challenges, such as a lack of adequate infrastructure, accessible educational materials, and an insufficient number of teachers trained and specialized in special education (21, 22).

The tendency to integrate children with visual impairments into regular schools without specific adaptations reduces the effectiveness of their learning. The teaching process through activities, strategies, or organizational methods can also constitute a barrier to learning. The participation of indigenous and migrant students with visual impairments is hindered when the starting point is a homogeneous view of the student body, particularly when they are blind to diversity. Eliminating this barrier involves addressing the educational needs of the entire group, approaching content in a differentiated and diversified manner, considering that each individual's learning styles, times, and rhythms are different, and recognizing that not everyone learns in the same way or at the same speed, or with the same teaching strategies, and that this can create an environment that does not favor their academic and social development.

Low educational attainment, combined with prejudice and discriminatory attitudes, often leads to early school leaving and dropout, thereby reducing these children's chances of accessing higher education, which would enable them to integrate into the labor market fully. This creates social vulnerability that contributes to cycles of poverty and exclusion, affecting the self-esteem and autonomy of people with visual impairments.

The indirect economic impact of these conditions is reflected in recurring expenses such as transportation to access specialized services, home adaptations, or changes in the school environment, all focused on ensuring the safety and autonomy of minors. Added to these costs are those derived from medical treatments and therapies. The need for personalized accompaniment and constant support reduces the economic productivity of family members, who often have to take on caregiving roles, limiting their ability to participate in the labor market (23, 24). At the macroeconomic level, this situation leads to a loss of human capital. It exacerbates regional and social inequalities, affecting both national economic development and social cohesion in the surrounding area.

## 3 Political participation in the challenges of prevention and comprehensive care

In Mexico, there are more than 7,650,000 people with some form of disability, of whom 47.3% are older adults and 34.8% are between 30 and 59 years old (25). It can therefore be established that most of them currently meet the requirements to exercise their rights and obligations of political participation. In this regard, political participation is seen as a crucial factor that can transform the current approach to congenital eye malformations in Mexico. Unfortunately, the lack of public policies addressing the systematization of prevention, diagnosis, and specialized treatment for these pathologies hinders progress in reducing preventable visual impairment. It is necessary to strengthen and expand access to neonatal screening programs, which aim to identify congenital eye malformations promptly, as well as to create care networks that integrate a multidisciplinary team (ophthalmologists, geneticists, pediatricians, and social workers) (26). Continuing education for medical personnel and community awareness are essential tools that could increase detection of these conditions, with a view to reducing environmental risk factors.

Public education campaigns should be promoted, targeting women of reproductive age and their families, informing them of the importance of prenatal care and the risks associated with specific exposures. The involvement of civil society, patient associations, and non-governmental organizations should improve and prioritize this issue with the authorities (27). The proper allocation of economic resources and the formulation of focused, evidence-based policies will make it possible to establish a regulatory framework that promotes research, technological development, and access to cutting-edge treatments for congenital eye malformations.

Below are two examples of public policies that offer a structured approach combining the integration of existing child health services with legislative support, which could be considered to adapt to the cultural and social context in Mexico. The first example comes from Tanzania, where eye conditions were incorporated into the Integrated Management of Neonatal and Childhood Illnesses program in 2019. This program is an algorithm-based, symptom-oriented approach in which primary health care workers perform a structured assessment of the child, leading to the classification of the condition and its severity, and a specific care plan with guidance on how to advise the mother (28). Mexico could replicate this model to include more robust detection and timely follow-up of eye malformations from the earliest levels of care, thereby strengthening surveillance and early prevention.

The second example focuses on Canada, where the passage of the National Vision Care Strategy Act (Bill C-284) in 2024 established a comprehensive legislative framework to improve access to eye health and vision rehabilitation services nationwide (29). This policy includes investment in research for treatments, promotion of education, and equitable access to vision care services.

## 4 Conclusions

Congenital eye malformations in Mexico represent a multidimensional challenge that affects not only the individual with the condition but also their family, given the significant financial burden associated with these problems. The increasing prevalence of these pathologies, coupled with limited capacity for diagnosis and timely treatment, requires coordinated action between the health sector and society to improve care or establish better prevention mechanisms. Although folic acid supplementation and visual screening are recognized as key to prevention and early detection, there are still communities with limited access to prenatal care due to sociocultural barriers.

Mexico's genetic diversity, resulting from the mixing of multiple indigenous and mestizo populations, offers a unique opportunity to study these pathologies in a broad and varied genomic context. Future studies should focus on generating up-to-date and comprehensive epidemiological data that reflect this genetic diversity and allow for more accurate identification of the specific genetic and environmental factors for the Mexican population. It is essential to strengthen the health infrastructure and train health professionals in these congenital defects, but above all, comprehensive public policies based on scientific evidence must be developed. The active participation of civil society is essential to overcome inequalities and thereby improve access to eye health services.
